# A Preliminary Investigation of Thermally Stable Schiff Base Metal Complexes for Hyperthermia: Synthesis and Biological Evaluation

**DOI:** 10.3390/antiox13121586

**Published:** 2024-12-23

**Authors:** Vigneswari Sankara Narayanan, Soven Dhawa, Amritha Sukumaran, Bharathi Hassan Ganesh, Jeya Rajendran, Kondapa Naidu Bobba, Prasanna Ramani

**Affiliations:** 1 Department of Chemistry, Loyola College, Affiliated to University of Madras, Chennai 600034, India; vigneswarinarayanan@gmail.com (V.S.N.); sovendhawa@gmail.com (S.D.); 2 Dhanvanthri Laboratory, Department of Chemistry, Amrita School of Physical Sciences, Amrita Vishwa Vidyapeetham, Coimbatore 641112, India; s_amritha@cb.students.amrita.edu (A.S.); hg_bharathi@cb.students.amrita.edu (B.H.G.); 3 Center of Excellence in Advanced Materials & Green Technologies (CoE–AMGT), Amrita School of Engineering, Amrita Vishwa Vidyapeetham, Coimbatore 641112, India; 4 Department of Nuclear Engineering, University of Tennessee-Knoxville, Knoxville, TN 37996, USA; kbobba@utk.edu

**Keywords:** antioxidant, degradation mechanism, kinetic parameters, transition metal complexes, hyperthermia

## Abstract

A novel Schiff base ligand (L), bearing N_2_O_2_ donor sites, was derived from the condensation of 5-chloromethylisophthaldehyde and phenylpropanolamine (PPA). Mononuclear Co(II), Cu(II), and Zn(II) complexes were synthesized and were characterized by FTIR, UV–Vis, ^1^H NMR, ESI-mass spectroscopy, molar conductance, and thermal and electrochemical studies. The thermal investigation revealed that the complexes were stable up to 150–250 °C and began to degrade in stages, resulting in the development of respective metal oxides. The Coats–Redfern integration method was used to calculate the kinetic and thermodynamic parameters, the energy of activation (Ea), and changes in enthalpy (∆H), entropy (∆S), and free energy (∆G) for each step of the degradation processes. For stage I decomposition, the calculated activation energy values of the complexes follow the order of Ea [Cu(L)] > Ea [Co(L)(H_2_O)_2_] > Ea [Zn(L)]. The influence of the temperature on the efficacy of antioxidant activities of the complexes with DPPH assay, ABTS assay, and hydroxy radical assay was investigated at various concentrations using ascorbic acid (AA) as the reference. Antioxidant activity was assessed at multiple temperatures to ascertain whether these complexes may be applied in radiation therapy enhanced with hyperthermia and found to be stable. Subsequently, the Cu(II) complex (C2) demonstrated a greater cytotoxicity (IC_50_ = 5.16 µM) than Co(II), Zn(II), and conventional cisplatin when in vitro cytotoxicity was evaluated against the MCF-7 cell line using the MTT method. Analyses of the thermal stability and ROS scavenging ability of complexes have demonstrated that these complexes have potential in hyperthermic radiation therapy.

## 1. Introduction

Schiff base metal complexes are gaining a greater role in cancer treatment due to their multiple anti-cancer and antioxidant properties. These complexes, formed by the condensation of primary amines with aldehydes or ketones, have structural flexibility and metal coordination capabilities, which increase their biological efficiency. These complexes’ distinct features, particularly their antioxidant [1] and redox activities, make them interesting candidates for combination cancer therapies, such as hyperthermia-enhanced radiation therapy. Hyperthermia, often known as heat treatment, is an adjuvant cancer therapy procedure in which the localized heating of tumors increases cancer cells’ sensitivity to radiation. Radiation therapy is one of the main cancer treatment modalities applied in 50–70% of cancer patients [2]. Despite the many advantages of this treatment, such as non-invasiveness, organ preservation, and spatiotemporal flexibility in tumor targeting, it can lead to complications in irradiated healthy cells/tissues. Hyperthermia has been demonstrated to increase oxidative stress in cancer cells, reducing their defenses and making them more vulnerable to radiation-induced damage. Cancer cells produce high levels of reactive oxygen species (ROS) [3,4], such as hydrogen peroxide, hydroxyl radicals, and superoxide anions, which can cause significant oxidative damage to cellular components such as DNA, proteins, and lipids. To maximize the synergistic effects of hyperthermia and radiation, medicines that control ROS levels are very desirable [5]. Schiff base metal complexes are useful for this purpose since they have both antioxidant and anti-cancer characteristics [6,7]. As antioxidants, these complexes can scavenge ROS and inhibit oxidative chain reactions, shielding healthy cells from radiation-induced oxidative damage while raising ROS levels in cancer cells [8]. The antioxidant properties of Schiff base metal complexes have been assessed by DPPH, ABTS, and hydroxy radical assays, which assess their ability to quench free radicals, donate electrons, and reduce oxidative stress. The therapeutic effects of Schiff base metal complexes in hyperthermia–radiation settings also depend on the metal ions within the complex, as certain metals significantly influence redox behaviors and biological activities [9,10]. For instance, complexes with Co(II), Cu(II), and Zn(II) have demonstrated strong ROS scavenging [11] and DNA binding capabilities [12,13], enhancing their potential as radiation-sensitizing agents. Furthermore, studies on Schiff base complexes with lanthanides, such as Dy(III) and Tb(III), have shown that the metal ion size and electronic properties affect ROS scavenging abilities, which may modulate the cellular redox balance in the presence of radiation [14,15].

This study involved the synthesis and characterization of Schiff base metal complexes with N_2_O_2_ donor sites, notably Co(II), Cu(II), and Zn(II) complexes. By investigating the thermal stability and reactive oxygen species scavenging activity of these complexes at different temperatures, a relationship between metal complex stability and antioxidant efficacy under simulated hyperthermic circumstances can be determined. This study revealed that Schiff base metal complexes can facilitate hyperthermia–radiation therapies by specifically regulating oxidative stress, therefore improving therapeutic results for cancer patients.

## 2. Materials and Methods

### 2.1. Chemicals and Physical Measurements

We purchased all the AR-grade reagents, commercially, for synthesis. We employed pre-coated thin-layer chromatography with silica gel to observe the reaction’s progression. We used a Buchii M-560 melting point apparatus (Buchii India Pvt., Ltd., Mumbai, India) to ascertain the melting points and a SysTronic digital conductivity meter (Systronics India Ltd., Ahmedabad, India) to evaluate the molar conductivity of the complexes. We employed a Perkin-Elmer CHN 2400 elemental analyzer (Perkin-Elmer (India) private limited, Thane, India) to assess the carbon, hydrogen, and nitrogen contents, and we determined the metal ion concentration gravimetrically as metal oxides. A Jasco V-730 spectrophotometer (JASCO Inc., Easton, MD, USA) was employed to obtain UV-visible spectra at ambient temperature. Shimadzu IR Affinity-1S system (Kyoto, Japan) was used to acquire the FTIR spectra and a JEOL-TOF JMS-T100LC (ESI) mass spectrometer (Jeol Ltd, Mumbai, India) for mass spectral analysis. We utilized the Gouy method to assess magnetic fields using a Sherwood Scientific magnetic balance and utilized the equation µ_eff_ = 2.828 (χm. T)^1/2^ B.M to determine the effective magnetic moments. In this context, χm represents the molar susceptibility adjusted with Pascal’s constants to account for the diamagnetism of all atoms within compounds. We utilized Bruker AMX-500 MHz and Bruker DPX-300 MHz spectrometers (Ettlingen, Germany) in deuterated solvents to acquire NMR spectra. We conducted electrochemical studies utilizing a Radiometer Analytical type PGZ 100, employing a conventional three-electrode configuration with a platinum wire auxiliary electrode and Ag/AgCl as the reference electrode [16]. We acquired the cancer cell lines from NCCS, Pune, and cultured the cells in MEM enriched with 10% FBS and penicillin/streptomycin antibiotics (0.5 mL^−1^), under a 5% CO_2_/95% air environment at 37 °C.

### 2.2. Synthesis of Co(II), Cu(II), and Zn(II) Complexes

The Schiff base ligand was synthesized with N_2_O_2_ donor sites, L = 2,2′-(((1,1′)-(5-(chloromethyl)-1,phenylene)bis(methaneylylidene))bis(azaneylylidene))bis(1-phenylpropan-1-ol) (4), from 5-chloromethyl isophthalaldehyde (3), which was obtained by treating 15 mL of isophthaldehyde (1) in ethanol; then, it was added to the mixture of concentrated 2N hydrochloric acid (150 mL) and formaldehyde in H_2_O (37%), and the reaction mixture was stirred for 24 h at room temperature to yield a reddish orange product of 5-(chloromethyl)isophthaladehyde (3) [17,18].

**Scheme 1:**
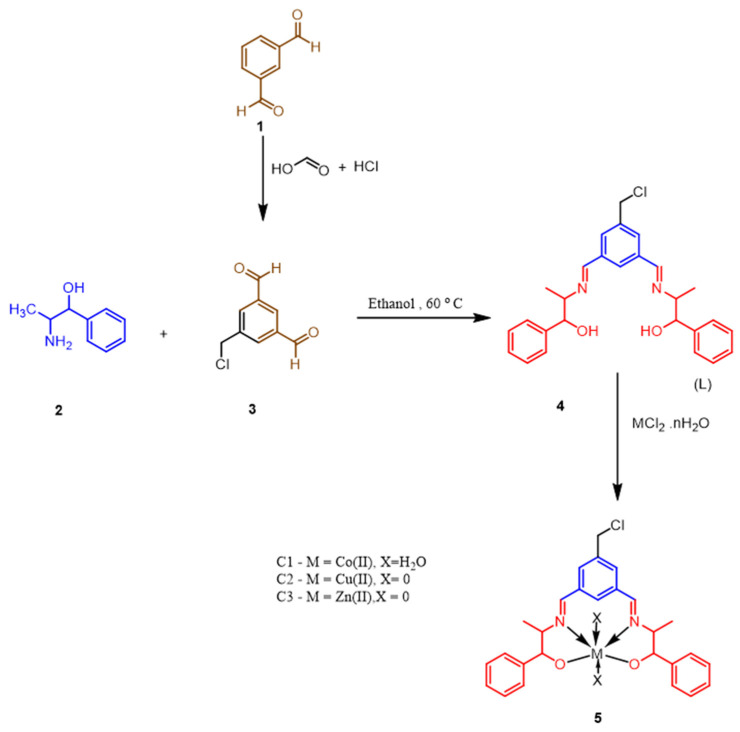
Synthesis of Co(II), Cu(II), and Zn(II) complexes with N_2_O_2_ donor ligand.

The product (3) was made to condense with two moles of phenylpropanolamine (2) in the ethanolic medium. The reaction was stirred for 8 h at 60 °C using a Dean–Stark apparatus and yielded a dark reddish brown solid product of Schiff base ligand, L (4)-bearing N_2_O_2_ donor sites. The progress of the reaction was monitored by TLC. After the completion of the starting material, the precipitated solid was filtered out, washed with diethyl ether, and dried under a vacuum. Recrystallization was performed in *n*-hexane to obtain the pure product. In an ethanolic medium, hydrated chloride salts of metal ions, such as Co(II), Cu(II), and Zn(II), were refluxed in a 1:1 ratio with the N_2_O_2_ donor ligand, L (4) (Scheme 1). The resulting complexes (C1–C3, 5) were filtered, washed with ethanol, then petroleum ether, and desiccated with CaCl_2_. The complexes were purified by column chromatography using petroleum ether and ethyl acetate (7:3) as eluents (Scheme 1).

### 2.3. Method Adopted forAntioxidant Assays 

The antioxidant activity of the samples was evaluated using DPPH, ABTS, and hydroxyl radical scavenging assays [19]. The ligand (L), complexes C1–C3, and ascorbic acid (AA, as standard) were subjected to antioxidant evaluation at different concentrations (12.5, 25, 50, 75, 100, and 200 µM) by increasing the temperature (25, 50, 75, and 100 °C) for over 30 min. The required amount of samples were dissolved in DMF (1 mL) and MeOH (1 mL) and incubated at the required temperatures for a period of 30 min, and it was used for antioxidant analyses.

The DPPH (1,1-diphenyl-2-picryl-hydrazyl) radical scavenging activity assay is a standard method for evaluating antioxidant activity. This rapid technique involves testing various concentrations (12.5, 25, 50, 75, and 100 µM) and temperatures (25, 50, 75, and 100 °C) of the complexes (C1–C3) in 1 mL DMF, mixed with 1.0 mL of 0.4 mM methanol solution of DPPH. After 30 min of incubation at various temperatures, the absorbance was recorded at 517 nm using a JASCO model V-UV-Vis spectrophotometer. The reduction in absorbance, as the DPPH solution decolorizes from deep violet to light yellow, indicates the radical scavenging activity. The experiment was conducted thrice, and the average absorbance was noted for each concentration. Ascorbic acid served as the reference standard, and a control sample was prepared without tested or reference compounds. The radical scavenging activity was calculated as IC_50_ (%) = [(A_o_ − A_t_)/A_o_] × 100, where A_t_ is the absorbance of the tested sample and A_o_ is the blank sample. The percent inhibition was plotted against the log concentration to obtain the IC_50_ value, with a lower IC_50_ indicating a greater antioxidant activity.

The procedure for performing the ABTS assay commences with the preparation of the ABTS stock solution by dissolving 7 mM of ABTS (2,2′-azino-bis(3-ethylbenzothiazoline-6-sulfonic acid)) in deionized water. To generate ABTS radical cations (ABTS•⁺), this solution was combined with 2.45 mM potassium persulfate and incubated in darkness at ambient temperature for 12–16 h. Before utilization, the ABTS•⁺ solution was diluted with phosphate-buffered saline to achieve an absorbance of approximately 0.70 ± 0.02 at 734 nm, as determined spectrophotometrically. For the assay, a predetermined volume of the test sample was combined with the ABTS•⁺ solution, and the reduction in absorbance at 734 nm was measured, which indicates the scavenging activity of the antioxidant against the ABTS•⁺ radical. We used various concentrations (12.5, 25, 50, 75, and 100 µM) and temperatures (25, 50, 75, and 100 °C) of the complexes (C1–C3).

The hydroxyl radical scavenging assay is a popular method for determining the antioxidant activity of substances by measuring their ability to neutralize hydroxyl radicals (•OH). The assay typically includes producing hydroxyl radicals using the Fenton reaction, where ferrous sulfate (FeSO_4_) combines with hydrogen peroxide (H_2_O_2_). The standard approach involves preparing a reaction mixture with 1 mM FeSO_4_, 1 mM EDTA, 10 mM H_2_O_2_, 2.8 mM deoxyribose, and the test sample in a buffer. The mixture is incubated at 37 °C for 30 min to allow hydroxyl radicals to react with deoxyribose and form thiobarbituric acid-reactive compounds (TBARSs). Following incubation, 1% thiobarbituric acid (TBA) and 2.8% trichloroacetic acid (TCA) are added to terminate the reaction and generate a pink chromogen with the degradation products of deoxyribose. The reaction mixture is then heated at 90–100 °C for 15 min and cooled, and the absorbance is measured at 532 nm using a spectrophotometer. We used various concentrations (12.5, 25, 50, 75, and 100 µM) and temperatures (25, 50, 75, and 100 °C) of the complexes (C1–C3) for this analysis.

### 2.4. MTT Assay

The MTT assay is a colorimetric method that quantifies the reduction of yellow 3-(4,5-dimethylthiazol-2-yl)-2,5-diphenyl tetrazolium bromide (MTT) by mitochondrial succinate dehydrogenase. The assay relies on the cell count and the assumption that dead cells or their byproducts do not reduce tetrazolium. The MTT enters the cells and is transported into the mitochondria, where it is converted to insoluble, dark purple formazan crystals. The cells are subsequently solubilized with DMSO and released, solubilized formazan reagent is quantified spectrophotometrically at 570 nm. Cell viability was assessed using the MTT assay through three independent experiments, each involving six concentrations of substances tested in triplicate. Cells were trypsinized and subjected to the trypan blue assay to determine the number of viable cells in the solution. Cells were enumerated using a hemocytometer and planted at a density of 5.0 × 10^3^ cells per well in 100 μL of media in a 96-well plate, followed by overnight incubation at 37 °C. Following incubation, the old media were removed and 100 µL of fresh media was introduced with varying concentrations of the test substance into the designated wells of the 96-well plates. After 48 h, the drug solution was discarded and fresh medication was introduced along with MTT solution (0.5 mg/mL) to each well, then the plates were incubated at 37 °C for 3 h. Upon the completion of the incubation period, precipitates are generated due to the conversion of MTT salt to chromophore formazan crystals by cells possessing metabolically active mitochondria. The optical density of solubilized crystals in DMSO was assessed at 570 nm using a microplate reader. The proportion of growth inhibition was determined using the subsequent formula:

$$\%\;inhibition=\frac{100\;(control-treatment)}{control}$$

The IC_50_ value was determined by using the linear regression equation Y = Mx + C, where Y = 50, and M and C values were derived from the viability graph.

### 2.5. Statistical Analysis

All studies were conducted in triplicate, and findings are expressed as the mean ± standard deviation (SD) of three replicates.

## 3. Results and Discussions

### 3.1. Physical Parameters

Mononuclear Co(II), Cu(II), and Zn(II) complexes (C1–C3), were found to be soluble in DMF and DMSO. The yield (%), color, melting points (°C), and *m*/*z* ratio for the synthesized compounds are presented in Table 1. The non-electrolytic behavior of complexes (in DMF, 10^−3^ M) was confirmed from molar conductance values (14.3–19.7 ohm^−1^ cm^2^ mol^−1^) [20], which further support the existence of metal and ligand in a 1:1 ratio and establish the stoichiometry as [ML(H_2_O)_2_] for the Co(II) complex and [ML] for Cu(II) and Zn (II) complexes, where the ligand, L, acts as a tetradentate-coordinating agent [20,21]. The proposed molecular formulae of the synthesized compounds were further confirmed by elemental analysis.

### 3.2. FT-IR Spectroscopic Analysis

**Figure 1:**
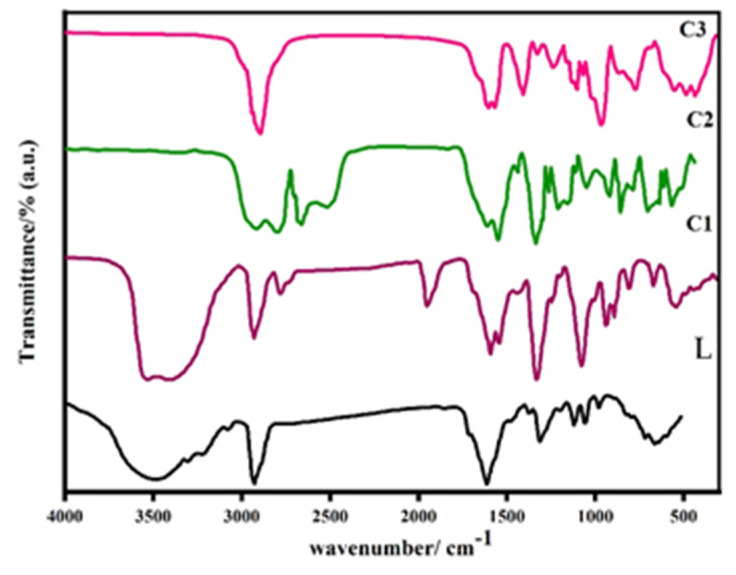
FTIR spectra of the ligand, L, and complexes, C1–C3.

The IR spectrum of the ligand (Figure 1) shows broad and strong peaks at 3498 cm^−1^, 3020 cm^−1^_,_ and 1692 cm^−1^, which are attributed to the stretching frequencies of ʋ(-OH), ʋ(ar.-C-H), and ʋ(-C=N) groups, respectively. The appearance of the ʋ(-C-Cl) peak at 1201 cm^−1^ renders the formation of the Schiff base ligand, L (Table S1) [18,22]. The shifting of ʋ(-C=N) towards the lower wavenumber region (1516–16,112 cm^−1^), which is the donation of the lone pair of electrons from azomethine nitrogen to the central metal atom, results in the formation of a coordinate linkage (M-N) [20]. The appearance of the -C-O peak at 1376–1401 cm^−1^ in the spectra of the complexes C1–C3 (Figure 1) indicates the deprotonating of ligands in bond formation with metal ions [22]. The appearance of new bands at 536–565 and 588–590 cm^−1^ provide evidence for the M-N and M-O bond formation in complexes, which further confirm the ligand, L, as a tetradentate donor agent. The broad stretching vibration peak at 3451 cm^−1^ and out-of-plane bending vibration peak at 824 cm^−1^, in the spectrum of complex C1 confirm the coordination of aqua ligand with the Co(II) metal ion [22,23].

### 3.3. Electronic Spectra and Magnetic Moment Analysis

**Figure 2:**
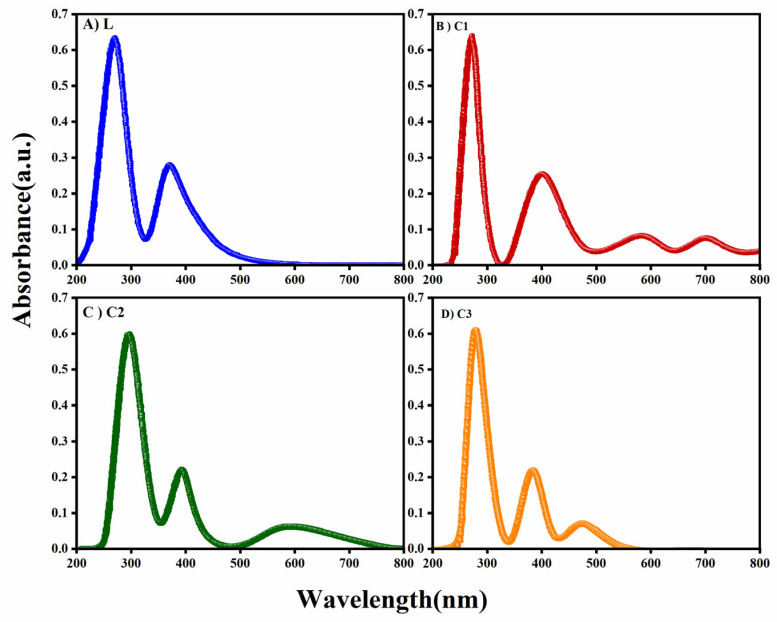
Electronic absorption spectra of (**A**) ligand, L, and (**B**–**D**) complexes, C1–C3 (DMF; 10^−5^ M).

Due to π → π* and n → π* intra-ligand transitions, the ligand L (Figure 2A) in DMF (10^−5^ M) displayed two very prominent bands in the 273 nm (36,630 cm^−1^) and 371 nm (26,954 cm^−1^) areas of its electronic spectra. The n → π* transitions were pushed towards 385–404 nm (24,752–25,974 cm^−1^) upon complexation, confirming the coordination between the metal ion and ligand, L [22,23]. Two absorption bands in the visible range of 17,152 cm^−1^ and 14,245 cm^−1^ were observed in the complex of Co(II), C1 (Figure 2B). These bands are associated with the ^4^T_1_(F) → ^4^A_2_g(F) (ϋ3) and ^4^T_1_g(F) → ^4^T_1g_(P) (υ_2_) transitions [24,25]. It was discovered that the calculated Racah parameter (B) for complex C1 (713 cm^−1^) was lower than the free ion value (1120 cm^−1^) [26]. This finding might be caused by the overlap of the ligand and metal d-orbitals. A significant degree of covalency between the metal–ligand links is indicated by nephelauxetic values (β) that fall between 0.68 and 0.92. The complex’s β values, C1 (0.68), confirmed the covalent nature of the bonds between the metal and ligand [26]. In addition, Co(II) ion pairing energy (P) is 22,000 cm^−1^ [27], greater than the complex’s ∆ _o_ C1 (12,834 cm^−1^) value. Consequently, ∆ _o_ < P denoted the rise in high-spin, octahedral geometry. The Co(II) complex’s observed magnetic moment value (Table 2) of C1 (4.81 BM) provided additional evidence for the presence of a high spin and octahedral shape [27]. Broadband was observed at 16,778 cm^−1^ for complex C2 (Figure 2C), supporting the square planar shape [22] and generated by the ^2^B_1g_ → ^2^A_1g_ transition with a magnetic moment value of 1.83 BM. The charge transfer transitions of MLCT and square planar geometry are responsible for the absorption band at 21,186 cm^−1^ observed in the electronic spectra of the diamagnetic Zn(II) complex, C3 (Figure 2D) [28].

### 3.4. Mass Spectral Analysis

The mass spectrum of ligand L (Figure S1) shows a molecular ion peak at *m*/*z* 449, which corresponds to the molecular formulae of the proposed structure, [C_27_H_29_N_2_O_2_Cl]^+^. A series of fragmentation peaks were observed at *m*/*z* 270, 136, and 179, respectively. The molecular ion peaks for the ligand (Figure S1), complexes, C1 (*m*/*z* 543; Figure S2), C2 (*m*/*z* 513; Figure S3), and C3 (*m*/*z* 514, Figure S4) supported the proposed structure and showed a prominent fragmentation peak of *m*/*z* 270 and *m*/*z* 136, due to [C_18_H_20_O_2_] and [C_9_H_11_O], respectively, with the strongest base peak at *m*/*z* 179, corresponding to [C_9_H_7_N_2_Cl]^+^.

### 3.5. H-NMR Spectroscopic Analysis

The ^1^H-NMR spectrum of ligand L (Figure S5) using DMSO-d6 at room temperature and tetramethysilane (TMS) as internal standard (Table S2) showed a multiplet in the region of δ = 7.10–8.02 ppm and singlets at δ = 1.66 (6H), 2.5 (2H), 13.17 (2H), and 3.73 (2H) ppm, corresponding to aromatic, two methyl protons = N-CH< and -CH(Ph)OH, and chlorobenzyl protons (Table S2). The presence of two imines and -OH protons was confirmed by singlet peaks at δ = 8.82 and 5.11 ppm, respectively, confirming the formation of ligand L. In the complex C3 (Figure S6), the disappearance of the -OH proton and the upfield shift of imine proton signals confirmed the coordination of these groups with the metal ions [23].

### 3.6. TG Analysis

**Figure 3:**
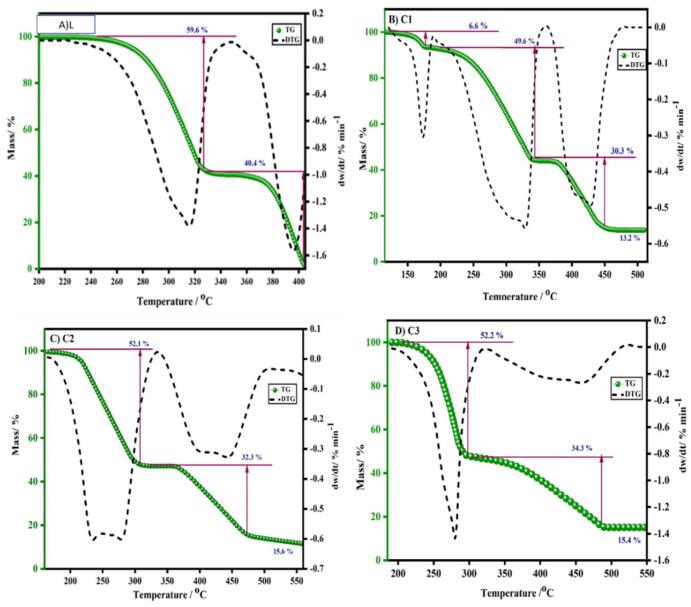
TG-DTG curves of the (**A**) ligand L and (**B**–**D**) complexes, C1–C3.

Thermograms of the ligand (L) and complexes C1–C3 were carried out in the range from 40 °C to 1100 °C, which indicated that the complex C1 was stable up to 150 °C, whereas the complexes C2 and C3 were stable up to 200 to 250 °C. The different stages of decomposition with the corresponding range of temperature and the experimental/calculated percentage of the mass of the compounds are summarized in Table S3. The thermal degradation of the ligand L (Figure 3A) occurred with the first stage of decomposition in the region of 258–324 °C (centered at 314 °C) due to the loss of two molecules of the phenyl propanol group, and the second stage of decomposition took place at above 324 °C (centered at 394 °C), which corresponds to the loss of the remaining part of the disubstituted imine groups in the chloro benzyl moiety.

The thermogram of the complex C1 (Figure 3B) showed four stages of decomposition. Initially, the decomposition started at 150–180 °C (centered at 170 °C), which is due to the loss of coordinated diaqua ligands [29]. The second stage of decomposition occurred in the region of 181–340 °C (centered at 190 °C), with the loss of two molecules of phenyl propanol group. The third stage of decomposition was at 341–450 °C (centered at 394 °C), which corresponds to the loss of the remaining part of the disubstituted imine groups in the chlorobenzyl moiety. The final stage of decomposition ended with the formation of metal oxides (CoO). The thermograms of complexes (C2 and C3) (Figure 3C,D), with no peaks below 200 °C and a broad degradation step, indicated the absence of water molecules in the lattice or the coordination sphere. The first stage degradation of the complexes C2-C3 started in the region of 200–320 °C due to the loss of two molecules of the phenyl propanol group followed by the decomposition of the remaining part of the disubstituted imine groups of chlorobenzyl moiety in the region of 341–450 °C (centered at 394 °C). The final stage of decomposition ends with the formation of the metal oxides (CuO and ZnO).

### 3.7. Kinetics and Thermodynamic Parameters of Complexes C1–C3 

The explanation for both the non-spontaneity and thermal stability of complexes C1–C3 was greatly aided by the kinetic and thermodynamic parameter studies. Activation energy (Ea), enthalpy of activation (∆H), frequency factor (A), entropy of activation (∆S), and Gibbs free energy change in the decomposition (∆G) are the kinetic and thermodynamic parameters of complexes that were graphically obtained from TG and DTG data using the Coats–Redfern [30,31] Equation (1).

ln[−ln(1 − α)] = ln ART^2^/βE_a_ − E_a_/RT (1)

**Figure 4:**
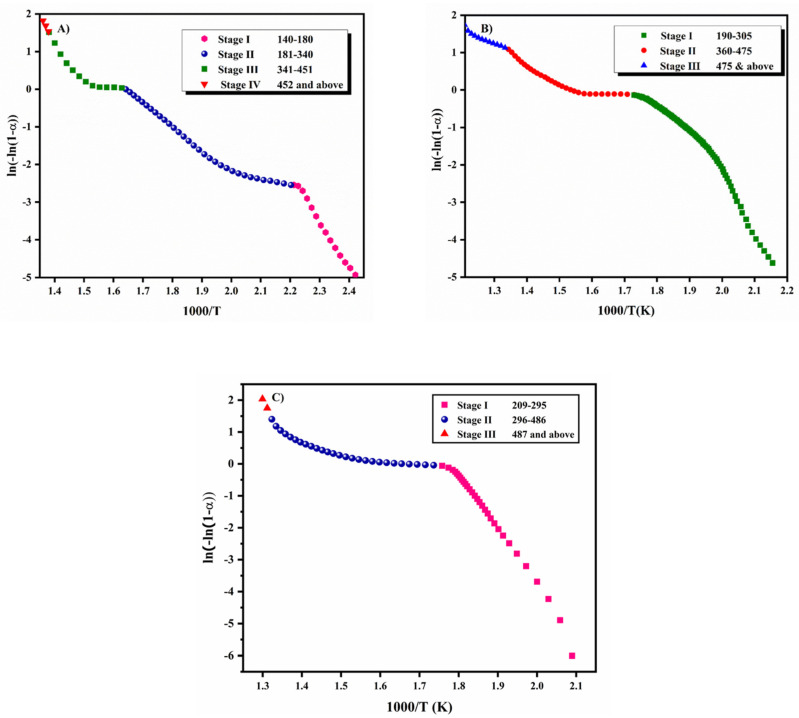
Coats–Redfern plot of ln[−ln(1 − α)] vs. 1000/T of the complexes: (**A**) = C1 at 10 °C min^−1^ in a N_2_ atmosphere; (**B**) = C2 at 10 °C min^−1^ in a N_2_ atmosphere; And (**C**) = C3 at 10 °C min^−1^ in a N_2_ atmosphere.

α = w_o_ − w_t_/w_o_ − w_f_ (2)

where W_o_ and W_f_ are the initial and final weights of the reaction, respectively, and W_t_ is the sample weight at a given temperature. The activation energy, universal gas constant, pre-exponential factor, and heating rate (10 °C/min) are also represented as the values of Ea R, A, and β. Plotting the curve of ln[ln(1 − α)] vs. 1000/T (Figure 4) generated the activation energy for each stage of decomposition, and a basic thermodynamic equation [32,33] was used to estimate other thermodynamic parameters.

∆S = 2.303R log (Ah/kT)(3)

where h and K are the Planck and Boltzmann’s constants, respectively.

∆H = E_a_ − RT (4)

∆G = ∆H − T∆S (5)

The decomposition of all complexes showed a best fit for first-order kinetics in all stages [34]. The thermo-kinetic data are summarized in Table S4. The high value of activation energies illustrated the high stability of complexes [35] and followed the order of E_a_ [Cu(L)] (**C2**) > E_a_ [Co(L)(H_2_O)_2_] (**C1**) > E_a_ [Zn(L)] (**C3**). The non-spontaneous degradation behaviors of the complexes were supported by the high positive values of ∆G, which suggest the high thermal stability of the complexes [36]. The negative values of the entropy change for all stages of decomposition correspond to the more ordered arrangement in complex formations, which suggests that the decomposition of the reactions is slower than the normal and non-spontaneous natures of the reaction [37]. The high positive ∆H values indicate the decomposition process of the complexes is endothermic in nature [38].

### 3.8. EPR Spectral Analysis

**Figure 5:**
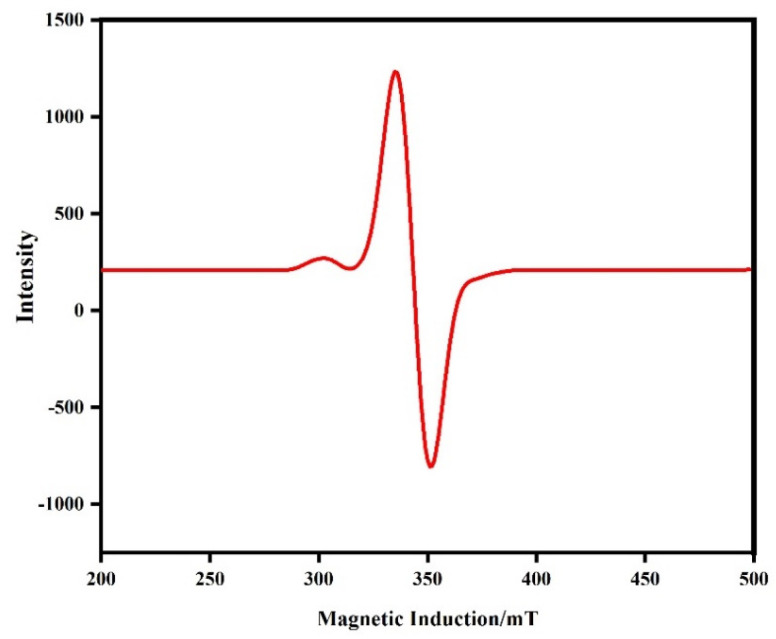
EPR spectrum of the complex C2.

The X-band EPR spectra of the complex C2 (Figure 5) were obtained in a DMSO medium at ambient temperature, utilizing a frequency of 9.4 GHz and a magnetic field strength of 3200 G. Tetracyanoethylene (ge = 2.00277) served as the standard. The EPR parameters (Table S5) of g‖, g⊥, A‖, and the energies of the d-d transition were computed to determine the molecular orbital coefficients, which encompass α2 (covalent in-plane σ-bonding), β2 (covalent in-plane π-bonding), and δ2 (covalent out-of-plane π-bonding), thereby elucidating the environment of a copper metal ion, including its geometry and the extent of covalency in the bonding between the metal and ligand.

The complex C2 (1.80 BM, Table 2) was found to be mononuclear, with µ_eff_ = µ spin. We calculated the magnetic moment value (2.08) from the giso value and found that the ligand completely stops the orbital magnetic moment. The spin Hamiltonian parameter showed the trend of g‖ > g⊥ > ge, which confirms the unpaired d electron of the complex C2 (2.24 > 2.037 > ge) is localized in the dx^2^ − y^2^ orbital, signifying the attributes of square planar geometry [22,39,40]. The geometric parameter, the G value for the complex C2 (7.55), exceeded 4, indicating that the exchange interaction in the solid state is minimal [41]. The computed g‖ value is 2.24, which is smaller than 2.3, indicating the covalent character in the metal–ligand bond [42]. The square planar geometry surrounding the Cu(II) ion primarily accounts for the co-factor value of the degree of geometrical distortion (f|| = 135.80 cm^−1^). The computed value of α2 ranged from 0.386 to 0.740, indicating that the complex C2 (0.74) exhibited a covalent character between the metal and ligand. The computed β2 (0.76) and γ2 (0.41) values for the complex C2 were below 1.0, indicating that π-bonding possesses have a complete covalent character [22,43]. The computed value of K2║ (0.56) surpassed that of K2⊥ (0.39), thereby confirming the presence of out-of-plane bonding between the metal and ligand in a square planar configuration, in line with the results of electronic absorption studies [41,44].

### 3.9. Electrochemical Behaviour of Complexes

**Figure 6:**
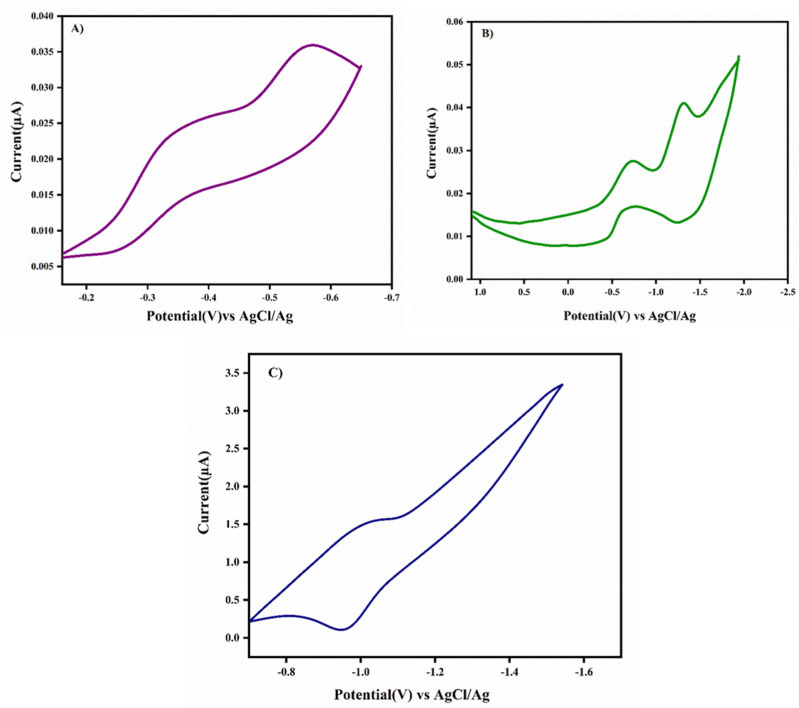
Cyclic voltammogram of the complexes (**A**) C1, (**B**) C2, and (**C**) C3 were examined at 10^−3^ M in DMF with tributoxyethyl phosphate (TBEP).

A cyclic voltammogram provides information about the redox behavior of complexes. The electrochemical characteristics of every complex (10^−3^ M) were examined in DMF with tributoxyethyl phosphate (TBEP) acting as a supporting electrolyte to control the chemical reaction pathway through an electron transfer mechanism (Table S9). By using a reference electrode of Ag/AgCl and a scanning rate of 100 mV s^−1^, the potential of cyclic voltammetry was measured between −4.0 and 1.0 V. The Co(II) complex C1 (Figure 6A) exhibited two redox processes in its electrochemical characteristics due to the Co(II)/Co(I) pair that was formed at E_pc(1)_ = 0.59–0.68 V and Epa(1) = 0.68–0.76 V. (E_p(1)_) = 0.08 V and i_pa(1_)/i_pc(1)_ = 0.048, the ratio of anodic to cathodic peak current, indicate that this coupling is reversible. The anodic peak at E_pc(2)_ = −0.55–1.0 V with (E_p(2)_) = 0.94 V and the second redox peak of Co(I)/Co(0), which are indicative of the Co(I)-Co(0) pair, can be found at E_pa(2)_ = −0.50 to −0.98 V. The peak current ratio i_pa(2)_/i_pc(2)_ = 0.69 indicates that the process represents a quasi-reversible two-electron transfer mechanism [45]. The redox behavior of the Cu(II) complex C2 (Figure 6B) showed two well-defined reversible redox processes. The first reduction peaks were found in the range of E_pc(1)_ = 0.028–0.042 V, with corresponding oxidation peaks in the range of E_pa(1)_ = 0.093–0.108 V. The peak separation (∆E_p1_) of this couple was 0.065 V. The ratio of anodic to cathodic current peak (i_pa1_/i_pc1_) was 1.25 and the second reduction was found in the range of E_pc(2)_ = (−0.97)–(−1.13) V, with a corresponding oxidation peak in the range of E_pa(2)_ = (−0.53)–(−0.96) V. The peak separation (∆E_p2_) of this couple is 0.44 V. The i_pa2_/i_pc2_ value falls at 0.95 V. Increasing the current density peak separation further indicates that the complexes are subject to quasi-reversible one-electron transfer each [46]. The voltametric measurement of the complex C3 (Figure 6C) showed a well-defined reversible redox peak, with E_pc_ = −0.047 to −0.069 V and E_pa_ = 0.11 to −0.25 V. The peak separation of this couple is ∆Ep = 0.063 V. The equivalent current in the cathodic and anodic peak (i_c_/i_a_ = 1.21) for these complexes indicates a reversible process with two electron transfers [47].

### 3.10. Antioxidant Studies

**Figure 7:**
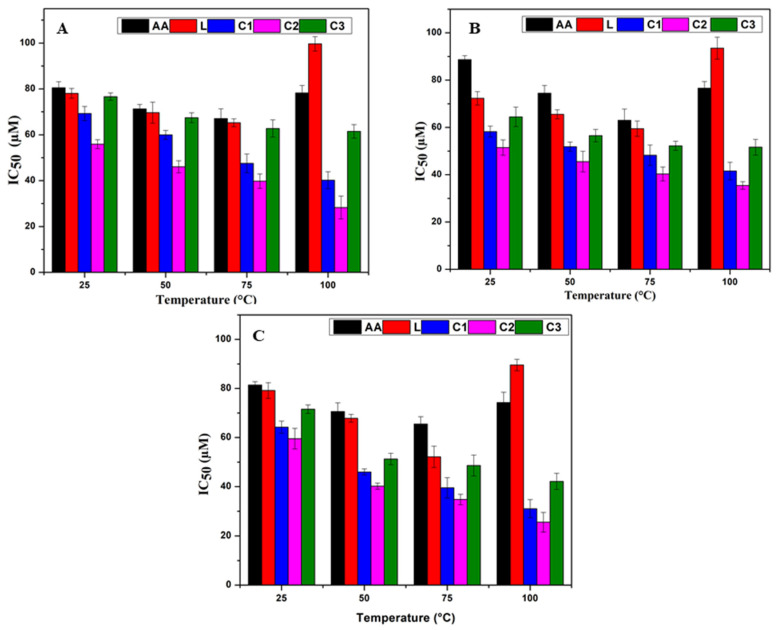
Plot of the IC_50_ values of the efficiency of the antioxidant activities of the complexes against different temperatures. (**A**) DPPH assay. (**B**) ABTS assay. (**C**) Hydroxyl radical assay.

The antioxidant activity of all the synthesized compounds were estimated using the DPPH, ABTS, and hydroxyl radical assays [19]. By using a linear regression analysis, we measured the antioxidant activities in terms of the IC_50_ value, or the effective concentration at which 50% of the radicals were scavenged (Tables S6–S8). The complexes C1–C3 and ligand (L) were shown to cause an increase in the scavenging abilities in a dose-dependent way (Figure 7) in all the assays. In the DPPH assay, all the complex exhibited a similar pattern, with the antioxidant behavior increasing with the increase in the temperature. The complex C2 was found to show a higher activity compared to the other complexes, with the IC_50_ value ranging from 40 to 50 µM at the various temperatures studied. The complex C3 was found to be the least active (IC_50_ = 58 to 62 µM); all the complexes exhibited a better antioxidant behavior than ascorbic acid (IC_50_ = 64 to 84 µM). The other antioxidant assays, such as ABTS and hydroxyl radical, displayed a similar trend. In all the antioxidant assays and temperatures, the complexes were found to be active when compared to ligand (L). The antioxidant efficiencies of C1 to C3 were shown to be in the following order: C2 > C1 > C3 > AA. In general, the hyperthermia–radiation therapy is performed at a temperature that is slightly higher than body temperature; in this aspect, the complex C2, with the highly reducing nature of Cu(II), was found to be active at 50 °C by exhibiting IC_50_ values of 42, 40, and 42 µM for the DPPH, ABTS, and hydroxyl radical assays, respectively. The Zn(II) complex, C3, which is diamagnetic, exhibited a lower scavenging effect than the other complexes and comparable results with the standard ascorbic acid. These results suggests that the synergistic interaction of metal ions and ligands is essential for the development of an effective antioxidant.

#### Thermal Stress Versus Antioxidant Efficiencies

In hyperthermia, radiation therapy oxidative stress is developed in normal cells as a result of the ROS that are generated. To scavenge these ROS, an effective drug that is stable at higher temperatures is essential. Antioxidant assays, including the DDPH, ABTS, and hydroxyl radical assays, were performed for all metal complexes, C1–C3, as well as the ligand. The samples were dissolved in DMF/MeOH and incubated at various temperatures (25, 50, 75, and 100 °C) for over 30 min, prior to the dilution by varying the concentration (12.5, 25, 50, 75, and 100 µM) (Figure 7) [5,8,9]. The scavenging activities of the complexes at different temperatures were calculated in terms of IC_50_ (Table S6). The antioxidant efficiency of the Cu(II) complex, C2, was found to show a higher scavenging effect upon increasing the temperature compared to the Co(II) and Zn(II) complexes as well as the standard in a dose-dependent manner. The antioxidant efficiencies of the complexes, C1–C3, and the standard underwent degradation upon increasing the temperature (at 100 °C). The results of the antioxidant activity of the complexes complement the thermal analysis, which demonstrates the loss in molecular weight. While typical biological systems operate at physiological temperatures (~37 °C), there are certain biological or biomedical applications where elevated temperatures are relevant. For example, hyperthermia treatments in cancer therapy or certain industrial bioprocesses involving thermophilic organisms can involve conditions above physiological temperatures. In these contexts, it is essential to evaluate the stability and functionality of antioxidants at elevated temperatures, to check their stability and efficacy. Antioxidants that maintain activity under elevated temperatures could be particularly advantageous for use in high-stress environments or formulations where prolonged stability is required [48]. In our study, though all the metal complexes displayed stability and activity at elevated temperatures, in particular, the Cu(II) complex exhibited a good antioxidant activity at higher temperature.

### 3.11. Anti-Cancer Studies

All the synthesized complexes exhibited a good antioxidant activity at elevated temperatures. In order to understand the potential of these complexes against cancer cells, in vitro anti-cancer studies were performed. The MTT assay was performed using complexes C1–C3 in the MCF-7 cell line [49]. The results were compared to those of cisplatin, a well-known anti-cancer medication, after 48-h routine exposure. The cytotoxic effect of the complexes was examined by cell viability measurement at the following concentrations: 5, 10, 25, 50, and 100 µM. The IC_50_ values of the complexes C1–C3 showed significant action in inhibiting the proliferation of MCF-7 cell lines compared to the drug cisplatin. For each concentration, these tests were conducted on a triplicate dataset. The Cu(II) complex C2 (IC_50_ = 5.16 µM; Figure S7) showed a higher cytotoxicity compared to the other complexes, C1 (IC_50_ = 23.19 µM) and C3 (IC_50_ = 23.43 µM). Although cisplatin remains the most effective agent, with an IC_50_ = 4.19 µM, the Cu(II) complex, C2, demonstrated promising results, with an IC_50_ value close to that of cisplatin. C2 was found to show five times higher activity than C1 and C3, which may be due to the reducing nature of metal ions. From these results, it is evident that the complex C2 can be a lead compound to treat cancer patients undergoing hyperthermia–radiation therapy.

## 4. Conclusions

We synthesized a novel tetradentate Schiff base ligand (L), featuring N_2_O_2_ donor sites, utilizing substituted isophthalaldehyde and 2-phenylpropanolamine. We successfully synthesized Co(II), Cu(II), and Zn(II) complexes (C1–C3) using the corresponding metal salts and analyzed them using various spectroanalytical methods. The synthesized Zn(II) and Cu(II) complexes exhibited a square planar shape, while the Co(II) complex displayed a distorted octahedral shape and a high-spin state. The TGA analysis indicates that all complexes (C1–C3) exhibited thermal stability at high temperatures. The thermal analysis revealed the coordination of the Co(II) metal ion with two aqua ligands in compound C1. The redox behavior of all the complexes was assessed via cyclic voltammetry, revealing that the Cu(II) complex showed a well-defined reversible redox process. We determined the kinetic parameters of complexes C1–C3 using thermal analysis data and the Coats–Redfern methodology. The results demonstrate that the decomposition process of the complexes is endothermic, as shown by the positive ∆H values. The positive ∆G values suggested a non-spontaneous process, while the negative ∆S values indicated a more ordered, activated complex compared to the reactants. The high activation energy values of the complexes signified enhanced thermal stability, with the stability hierarchy being C2 > C1 > C3. The antioxidant efficiency of the complexes (C1–C3) augmented in a dose-dependent manner with the temperature. The complex C2 exhibited a more pronounced scavenging action in a temperature-dependent manner relative to C1 and C3. The cytotoxicity experiments showed that the complex C2 outperformed the other complexes in inhibiting the growth of human breast adenocarcinoma (MCF-7) cell lines. Copper can alter its oxidation state from Cu^2+^ to Cu^+^, rendering it an effective antioxidant due to its ability to eliminate free radicals and engage in redox processes. Since hyperthermia–radiation therapy generates reactive oxygen species (ROS) in normal cells, it is crucial to have an effective drug that can scavenge these free radicals and act as an antioxidant, thereby exhibiting an enhanced anti-cancer activity. These results of C2 are good indications of treatment for cancer and oxidative stress created by hyperthermia–radiation therapy, which is underway in our laboratory.

## Figures and Tables

**Table 1:** Physical parameters of the ligand, L, and complexes, C1–C3.

Sample	Molecular Formula	Color	Yield (%)	Melting Point (°C)	Elemental Analysis (%)				Mass Value (*m*/*z*)
					C Cal. (Found)	H Cal. (Found)	N Cal. (Found)	M Cal. (Found)	
**L**	C_27_H_29_ClN_2_O_2_	Reddish orange	79	135	72.2 (71.6)	6.5 (6.2)	6.2 (5.9)	-	449
**C1**	[Co(L)(H_2_O)_2_]	Reddish brown	86	>250	59.8 (58.3)	5.8 (5.9)	5.2 (4.9)	10.9 (10.3)	543
**C2**	[Cu(L)]	Dark green	89	>250	63.5 (62.4)	5.3 (5.4)	5.5 (4.3)	12.5 (12.7)	513
**C3**	[Zn(L)]	Pale yellow	73	>250	63.3 (62.9)	5.5 (5.7)	5.3 (5.0)	12.8 (12.7)	514

**Table 2:** Electronic spectral data, magnetic moment (µ_eff_), electronic spectral data of band assignments, and ligand field parameters for the complexes.

Samples	Intra-Ligand Transitions (cm^−1^)		d-d Transition (cm^−1^)		10Dq	ʋ_2_/ʋ_1_	B	β	β%	LFSE	µ_eff_ (BM)
	Π − π*	N − π*	ʋ_1_	ʋ_2_							
L1	36,630	26,954	**-**	**-**	**-**	**-**	-	-	-	-	-
C1	35,842	24,752	17,152	14,245	12,834	0.83	713	0.68	35	−9711	4.81
C2	36,496	25,510	16,778	-	-	-	-	-	-	-	1.80
C3	36,900	25,974	21,186	-	-	-	-	-	-	-	-

## Data Availability

Data are contained within the article and Supplementary Materials.

## Supplementary Materials

The following supporting information can be downloaded at: https://www.mdpi.com/article/10.3390/antiox13121586/s1.
