# Supplementary material for: A Preliminary Investigation of Thermally Stable Schiff Base Metal Complexes for Hyperthermia: Synthesis and Biological Evaluation
Source: Antioxidants (Basel). 2024 Dec 23;13(12):1586. doi: 10.3390/antiox13121586 (PMC11673609; doi:10.3390/antiox13121586)
Supplement: Supplementary file 1 [file antioxidants-13-01586-s001.zip › antioxidants-3201273-supplementary.pdf]

A Preliminary Investigation of Thermally Stable Schiff Base Metal Complexes for Hyperthermia: Synthesis and Biological Evaluation

Vigneswari Sankara Narayanan [1], Soven Dhawa [2], Amritha Sukumaran [3,4], Bharathi Hassan Ganesh [3,4], Jeya Rajendran [1*], Kondapa Naidu Bobba [5] and Prasanna Ramani [3,4*]

[1]*Department of Chemistry, Loyola College, Affiliated to University of Madras, Chennai-600 034, India*

[2] *Advance Research Institute, Dr. MGR Educational & Research Institute (Deemed to be University), Chennai-600 095, India.*

[3]*Dhanvanthri Laboratory, Department of Chemistry, Amrita School of Physical Sciences, Amrita Vishwa Vidyapeetham, Coimbatore 641112, India*

[4] *Center of Excellence in Advanced Materials & Green Technologies (CoE–AMGT), Amrita School of Engineering, Amrita Vishwa Vidyapeetham, Coimbatore 641112, India*

[5]*Department of Nuclear Engineering, University of Tennessee-Knoxville, Knoxville, TN 37996, USA*

**E-mail: JR: jeyarajendran@loyolacollege.edu; PR: r_prasanna1@cb.amrita.edu*

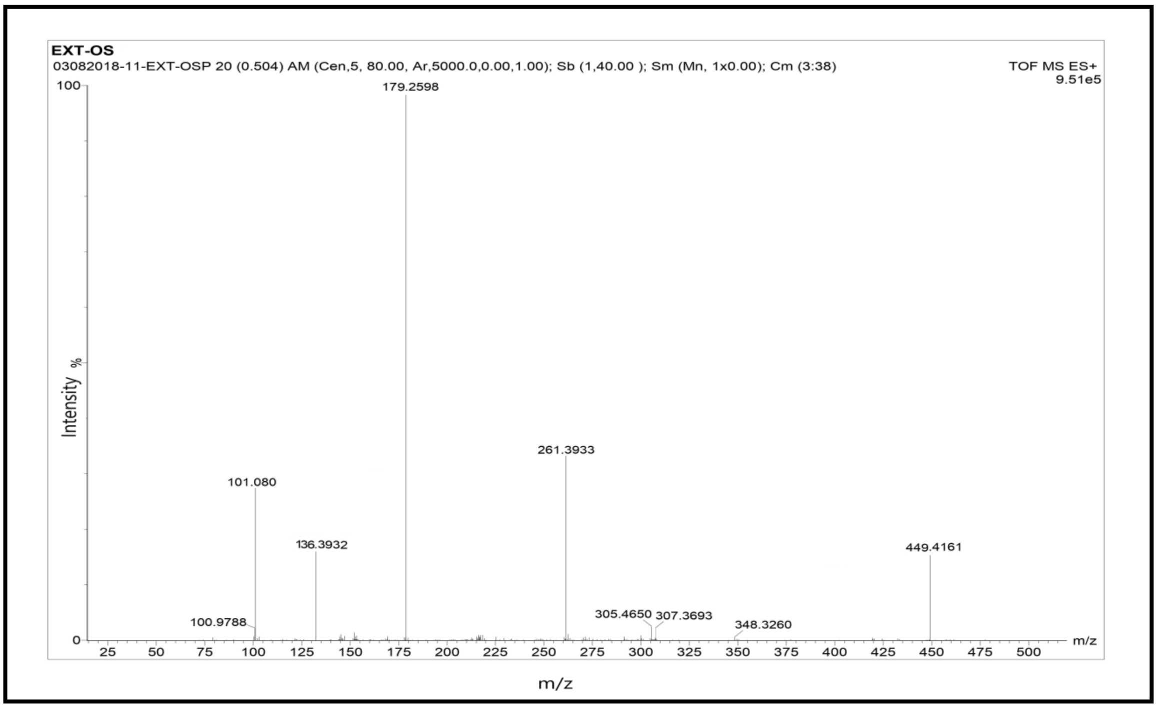

Figure S1.ESI-Mass spectrum of ligand, L

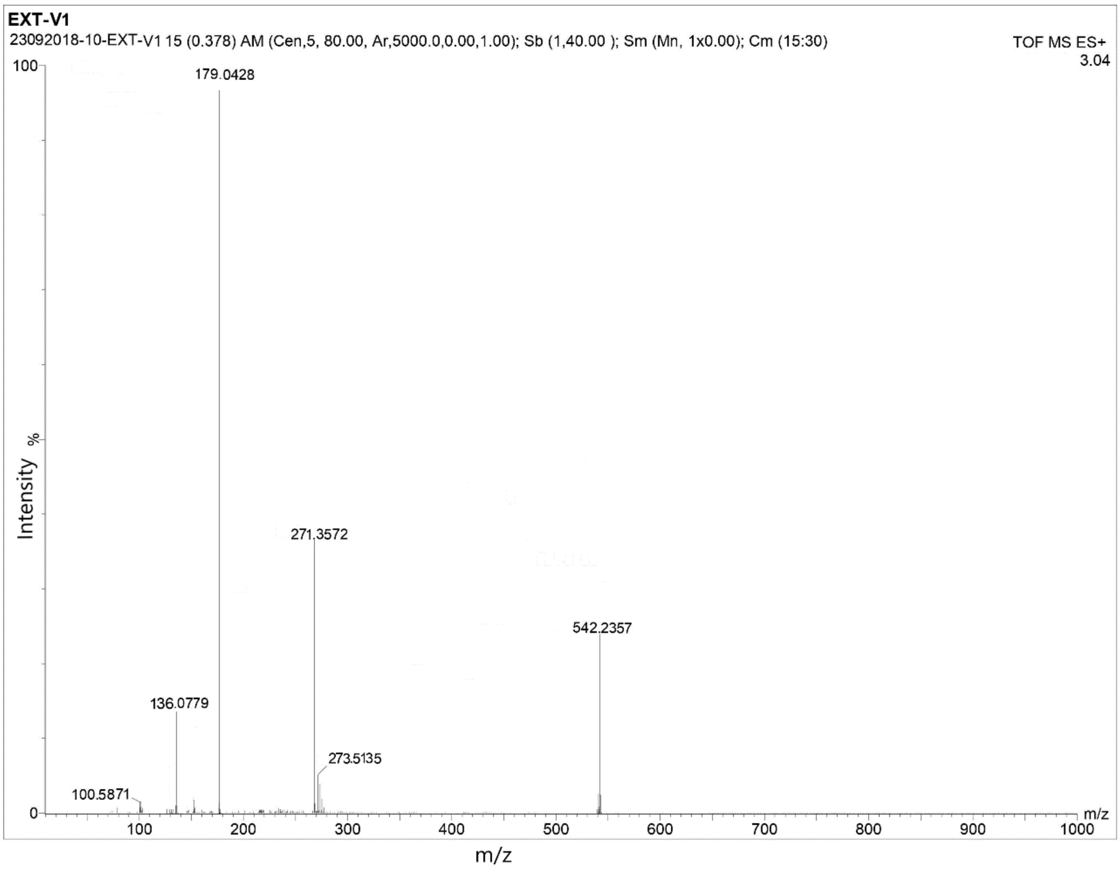

Figure S2.ESI-Mass spectrum of complex C1

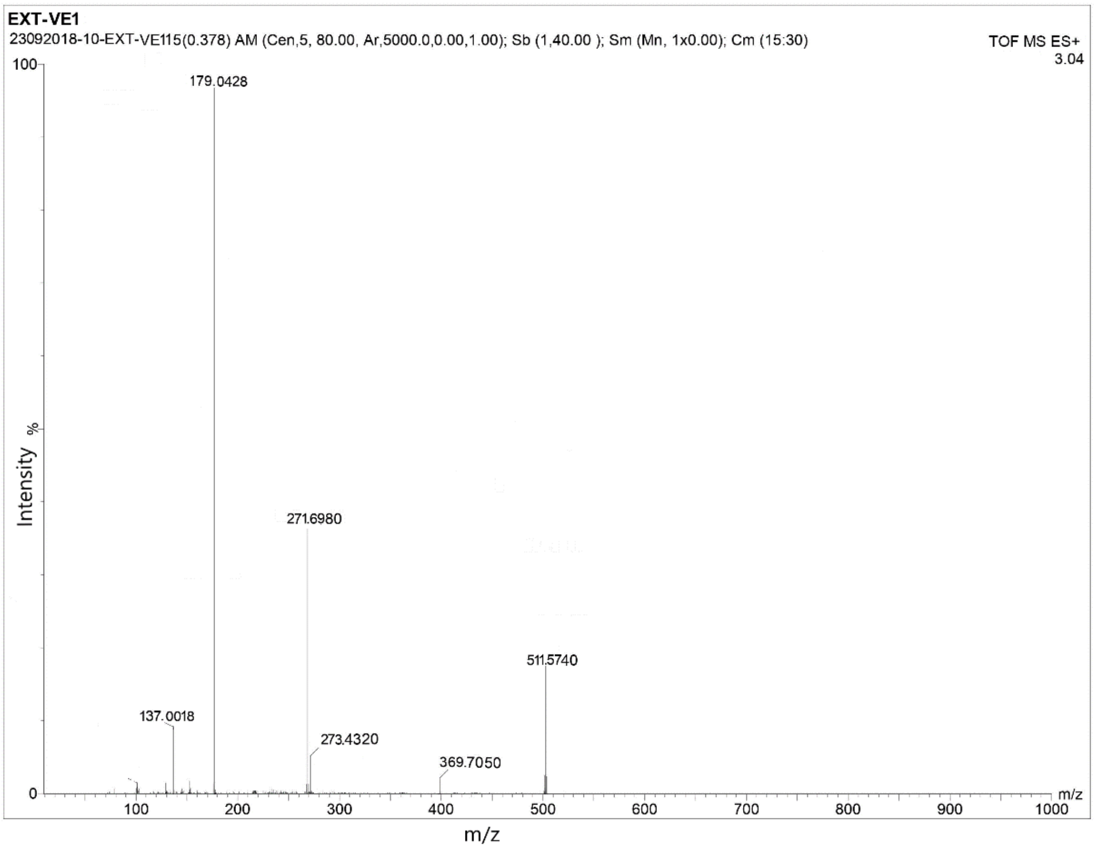

Figure S3. ESI mass spectrum of complex, C2

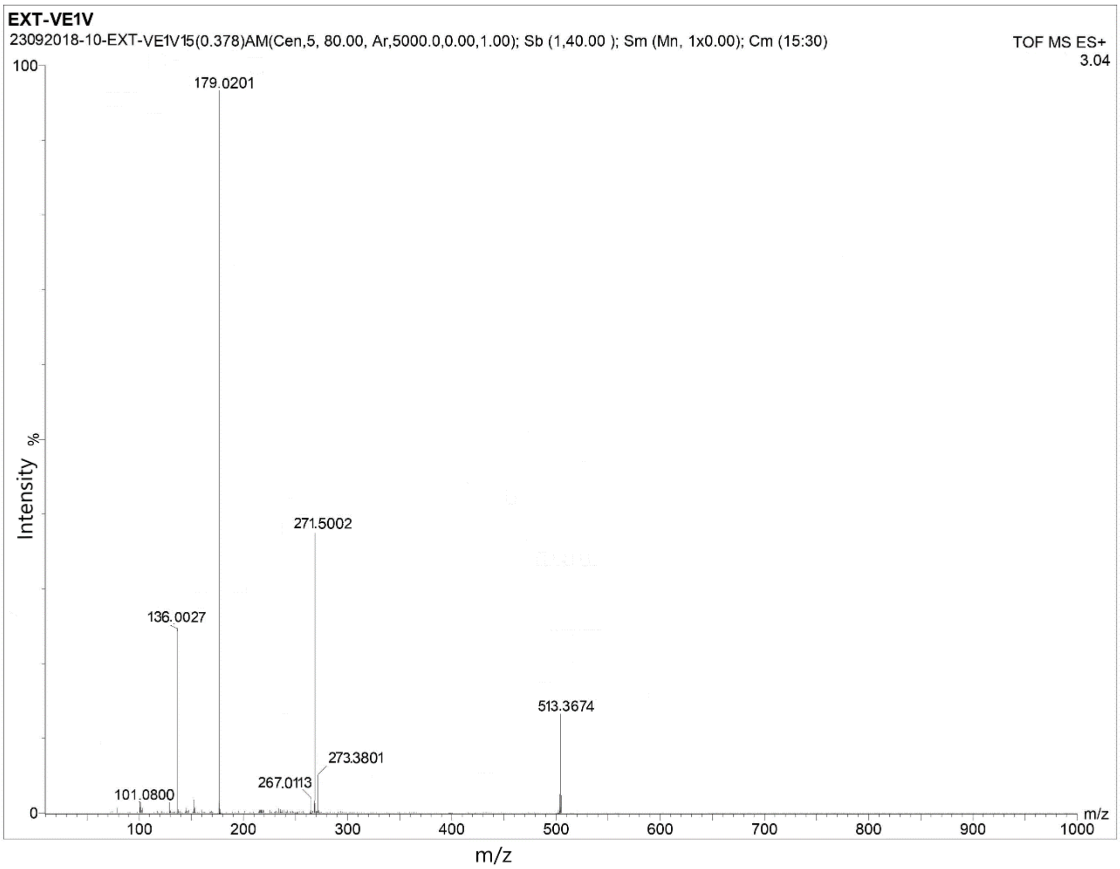

Figure.S4.ESI mass spectrum of complex, C3

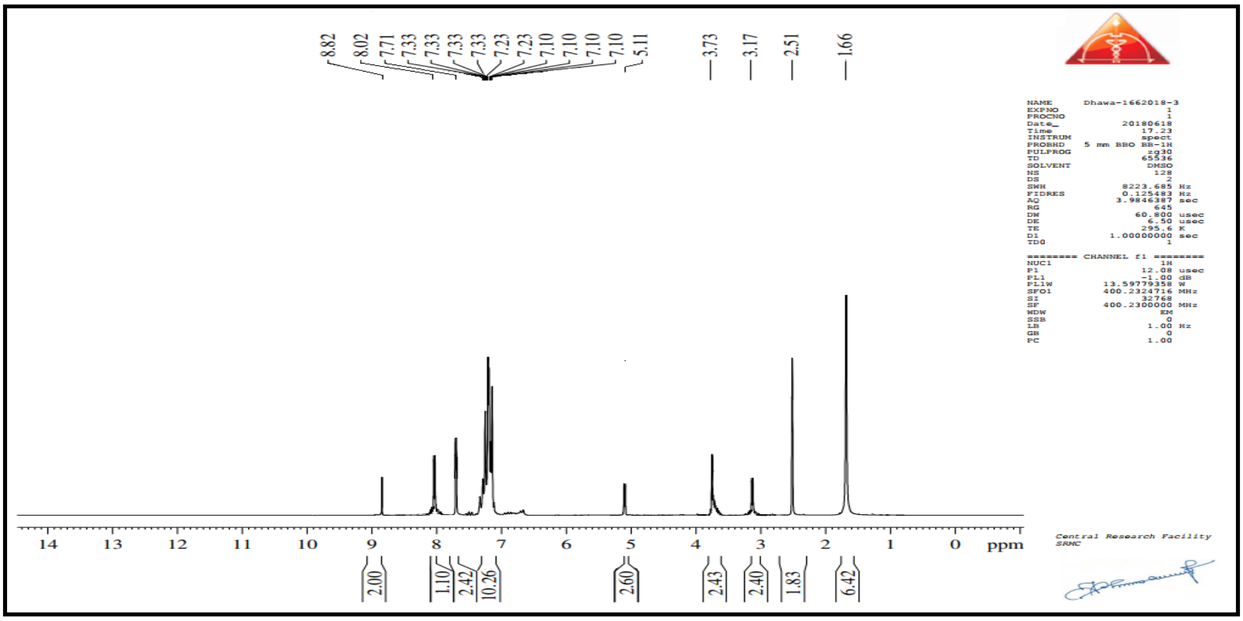

Figure.S5.^{1}H-NMR spectrum of ligand, L

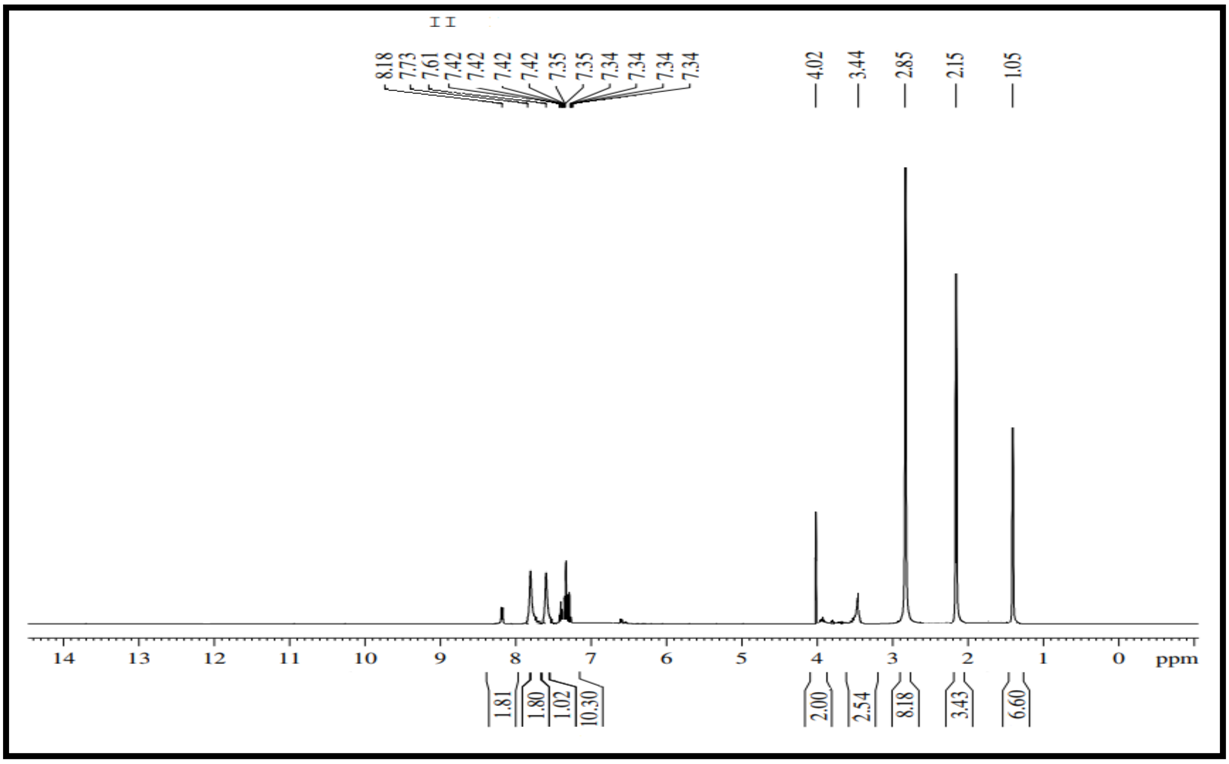

Figure.S6:^{1}H-NMR spectrum of complex, C3

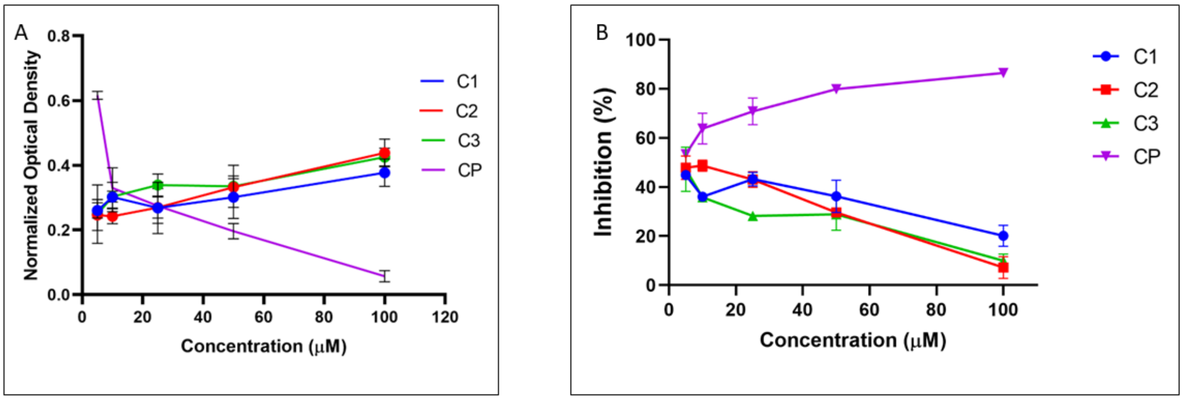

Figure.S7: A) Concentration-Normalised optical density curve B) Concentration-Percentage of inhibition curve of Anticancer studies. (CP: cisplatin)

Table.S1: IR spectral data of ligands, L and complexes C1-C3

Compounds	Wavenumber (cm^{-1})								
	-OH	Ar(-C-H)	(-C=N)	(-C-Cl)	(-C-O)	(-C-N)	(M-H_2O)	(M-N)	(M-O)
**L**	3498	3020	1692	1201	-	-	-	-	-
**C1**	3451	3025	1612	1214	1401	-	824	565	598
**C2**	-	3070	1516	1244	1376	-	-	542	588
**C3**	-	3100	1542	1258	1383	-	-	536	592

Table.S2: ^{1}H-NMR spectral data of ligand, L and complex, C3

**No.**	**^{1}H proton**	**Chemical shift value, δ (ppm)**	
		**L**	**C3**
1.	-HC=CH ar. (m)	7.1-8.02	7.17-7.91
2.	=N-CH(C**H_3**) (s)	1.66 (6H)	1.56 (6H)
3.	=N-C**H**(CH_3)-(s)	2.51 (2H)	2.71 (2H)
4.	>CH-C**H**(Ph)OH (s)	3.17 (2H)	3.51 (2H)
5.	Ph-C**H_2**Cl (s)	3.73 (2H)	3.73 (2H)
6.	=N-C**H** (s)	8.82 (2H)	8.18 (2H)
7.	-O**H** (s)	5.11 (2H)	-

Table.S3: Thermal data of ligand, L and complexes, C1-C3

**Samples**	**Decomposition stage**	**Temperature (°C)**	**Weight loss (%)**		**Inferences**
			**Theoretical**	**Experimental**	
**L1**	1	258-324	59.6	59.6	2[$C_9H_{11}O$]
	2	324 & above	40.4	40.4	$C_9H_7ClN_2$
**C1**	1	150-180	6.9	6.6	$2H_2O$
	2	181-340	49.3	49.6	2[$C_9H_{11}O$]
	3	341-450	32.5	30.3	$C_9H_7ClN_2$
	4	450 & above	13.8	13.2	CoO
**C2**	1	200-300	52.3	52.1	2[$C_9H_{11}O$]
	2	300-500	34.9	32.3	$C_9H_7ClN_2$
	3	500 & above	15.5	15.6	CuO
**C3**	1	230-313	52.19	52.2	2[$C_9H_{11}O$]
	2	314-501	34.8	34.3	$C_9H_7ClN_2$
	3	Above 501	15.7	15.43	ZnO

Table.S4:Kinetic and thermodynamic parameters of decomposition of the complexes, C1-C3 by Coats-Redfern method at 10ºC min^{-1} in N_2 atmosphere

Complexes	Steps	Ea ($J\ mol^{-1}$)	ΔH ($kJ.\ mol^{-1}$)	ΔS ($J.\ mol^{-1}.\ K^{-1}$)	ΔG ($J.\ mol^{-1}$)	R^2
**C1**	Stage 1	16.52	35.35	-128.66	52.817	0.99
	Stage 2	41.56	43.59	-288.86	14.95	0.98
	Stage 3	46.62	55.15	-286.81	18.64	0.99
	Stage 4	21.24	58.98	-45.44	27.49	0.99
**C2**	Stage 1	85.90	42.62	-184.50	92.27	0.99
	Stage 2	26.78	53.27	-313.45	20.39	0.98
	Stage 3	29.44	65.55	-312.87	24.18	0.97
**C3**	Stage 1	14.42	20.33	-82.30	19.54	0.99
	Stage 2	25.24	32.52	-319.81	12.28	0.85
	Stage 3	19.98	52.25	-54.96	28.67	0.97

Table.S5.EPR spectral parameters of complex, C2

Complexes	$g_{\parallel}$	$g_{\perp}$	so	$A_{\parallel}$	α^2	β^2	γ^2	$K^2_{\parallel}$	$K^2_{\perp}$	G	$f_{\parallel}$
C2	2.24	2.03	2.08	165×10^{-4}	0.74	0.76	0.41	0.56	0.39	7.55	135.8

Table.S6: IC_{50} value of the complexes and ligand with DPPH free radical at different temperature

Complex	IC_{50} value of the complex (μM)			
	**25 °C**	**50 °C**	**75 °C**	**100 °C**
**AA**	81±3	72±2	67 ±4	78 ±3
**L**	78 ±2	70±4	65 ±2	100±3
**C1**	69±3	60±2	48±4	40±4
**C2**	56±2	46±4	40±3	28±5
**C3**	77±1	67±2	63±4	62±3

Table.S7: IC_{50} value of the complexes and ligand with ABTS free radical at different

**Complex**	**IC_{50} value of the complex (μM)**			
	**25 ºC**	**50 ºC**	**75 ºC**	**100 ºC**
**AA**	89±2	74±3	63±5	77±3
**L**	72 ±3	66±2	60±3	93±5
**C1**	58±2	52±2	48±4	41±4
**C2**	51±3.	46±4	40 ±3	36±2
**C3**	64±4	57±3	52±2	52±3

Table.S8: IC_{50} value of the complexes and ligand with hydroxy radical at different temperature

**Complex**	**IC_{50} value of the complex (μM)**			
	**25 ºC**	**50 ºC**	**75 ºC**	**100 ºC**
**AA**	81±1	71±4	66±3	74 ±4
**L**	79±3	68±2	52±4	90±2
**C1**	64±2	46±1	40±4	31±4
**C2**	60±4	40±1	35±2	26±4
**C3**	72±2	51±2	49±4	42±3

Table.S9. Electrochemical data of the complexes, (C1-C3)

**Complexes**	**E_{pa1} (V)**	**E_{pc1} (V)**	**ΔE_{p1} (V)**	**i_{pa1}/i_{pc1}**	**E_{pa2} (V)**	**E_{pc2} (V)**	**ΔE_{p2} (V)**	**i_{pa2}/i_{pc2}**
**C1**	-0.248	-0.339	0.081	0.016	-0.494	-0.570	0.086	1.0
**C2**	-0.434	-0.630	0.166	0.71	-1.233	-1.317	0.084	0.2
**C3**	-0.965	-1.000	0.035	0.96				
